# *Pestivirus* infection in cattle dairy farms: E2 glycoprotein ELISA reveals the presence of bovine viral diarrhea virus type 2 in northwestern Italy

**DOI:** 10.1186/s12917-017-1305-z

**Published:** 2017-12-04

**Authors:** Chiara Nogarol, Nicola Decaro, Luigi Bertolotti, Barbara Colitti, Bryan Iotti, Stefano Petrini, Maria Stella Lucente, Gabriella Elia, Giovanni Perona, Margherita Profiti, Canio Buonavoglia, Sergio Rosati

**Affiliations:** 1 Department of Veterinary Science, University of Turin, Turin, Italy; 2 Department of Veterinary Medicine, University of Bari, Bari, Italy; 3 Istituto Zooprofilattico Sperimentale dell’Umbria e delle Marche, Perugia, Italy; 4 Largo Paolo Braccini, 2, 10095 Grugliasco, Italy

**Keywords:** Bvdv-1, Bvdv-2, HoBi-like *Pestivirus*, E2 glycoprotein, Bulk milk ELISA

## Abstract

**Background:**

Bovine viral diarrhea virus (BVDV) types 1 and 2 are members of the *Pestivirus* genus of the Flaviviridae family. This genus also includes the HoBi-like virus, tentatively classified as BVDV type 3. BVDV-1 is widely distributed in Italy despite the extensive use of BVDV-1-based vaccines, while BVDV-2 and HoBi-like *Pestivirus* have been detected occasionally. Monitoring the occurrence of sporadic or atypical pestiviruses is a useful approach to evaluate the need for additional vaccine strains that can be used in BVDV control programs.

**Results:**

In this study we developed a multiwell antibody ELISA based on the recombinant E2 protein of the three bovine pestiviruses. We evaluated the assay’s applicability for surveillance purposes using pooled milk samples, each prepared from a maximum of 35 lactating cows and collected from 176 dairy herds. As expected, the majority of the pooled samples reacted to a greater extent against the BVDV-1 E2 antigen. All three milk pools from a single farm reacted to the BVDV-2 antigen, however. Further analysis using spot tests, antigen detection, and sequence analysis of the 5′-UTR region confirmed the presence of five persistently infected calves carrying a BVDV-2a strain.

**Conclusions:**

This study highlights for the first time that sporadic circulation of BVDV-2 can be predicted by immunoenzymatic methods in the absence of specific vaccination.

**Electronic supplementary material:**

The online version of this article (10.1186/s12917-017-1305-z) contains supplementary material, which is available to authorized users.

## Background

Bovine viral diarrhea virus (BVDV) types 1 and 2 belong to the *Pestivirus* genus of the Flaviviridae family, along with border disease virus (BDV) and classical swine disease virus [1]. Other atypical pestiviruses include HoBi-like virus, tentatively classified as BVDV type 3 [2, 3], and wild ungulate pestiviruses. The severity of clinical signs is strictly associated with the viral strain and the type of infection. In most cattle infected transiently with BVDV, disease signs are mild, characterized by low-grade fever, diarrhea, and coughing [1, 4]. Infection is sustained by persistently infected (PI) carriers that acquire the infection with a non cytopathic (NCP) strain early in the fetal stage and remain immunotollerant virus shedders for life. The lifespan of PI animals is usually short due to early culling or the development of mucosal disease (MD), a fatal outcome arising from mutation of the NCP strain to its cytopathic (CP) counterpart.

The distribution of BVD virus types and subtypes is continuously evaluated in some European countries. A well-established procedure of sequencing the 5’UTR viral fragment from PI animals has been widely used, allowing characterization of the circulating viral strains [5]. BVDV-1 is detected with higher frequency than BVDV-2, but BVDV-2 outbreaks have been frequently associated with severe acute disease [6–8]. Up to 17 BVDV-1 subtypes have been detected in Italy over the course of the last 18 years despite the regular use of BVDV-1 vaccines for nearly two decades. The hypothesis is that some of the newly described subtypes may escape control through herd immunity [9]. In contrast, BVDV-2 is only partially neutralized by BVDV-1-induced immunity and has seldom been identified [7, 10].

Circulation of unusual or atypical pestiviruses has been linked to iatrogenic transmission, including the preparation of vaccines contaminated by NCP strain of BVDV type 2 [11]. The origin of the infection has not been clearly established in some recent reports, for example, the single outbreak of HoBi-like *Pestivirus* in southern Italy [3]. Circulation of BVDV strains that may either not or only partially be covered by vaccine-induced herd immunity raises major concerns [12], since the impact of infection is expected to be similar to that of a naïve population. In this light, monitoring of possible introduction of BVDV-2 and HoBi-like *Pestivirus* could provide a valuable tool to assess the effectiveness of BVDV-1-based vaccination programs and prevent the spread of viral strains. Unfortunately, genetic classification of BVDV strains in the course of PI identification is not routinely carried out by diagnostic laboratories, and analysis of BVDV isolates is often obtained years after the identification of PI animals, limiting investigation into risk factors for the introduction of such strains.

To our knowledge, the only serological diagnostic tool that can differentiate BVDV infections is the cross-seroneutralization test [13]. The E2 glycoprotein is the most divergent antigen between BVDV type 1, 2, and HoBi-like *pestivirus* and it is able to elicit neutralizing antibodies [14, 15]. Accordingly, we developed a multiwell antibody ELISA based on the E2 protein of the aforementioned viruses and evaluated, using pooled milk samples collected from dairy herds, the test’s applicability for surveillance purposes. A background of BVDV-1 infection and/or immunization clearly emerged, with greater reactivity against the BVDV-2 antigen noted in a single dairy herd. Further investigation using spot tests (sample of animals of a certain age [16]), individual milk samples, and antigen/RNA detection allowed the identification of several PI animals harboring the BVDV-2a subtype.

## Results

Sheep immunized with the inactivated whole virus preparation of BVDV type 1, 2, and HoBi-like *pestivirus* developed a strong immunoresponse against E2 viral glycoprotein (average of homologous virus-neutralization [VN] titers ranged from 445 to 2702). The homologous VN titer was at least three times greater than the heterologous VN titer (Table 1). The three control sera were normalized by diluting the two most reactive sera into negative bovine serum to attain a homologous VN titer equal to the less reactive serum. Checkerboard titration was performed to evaluate antigen expression levels. The results showed a large difference in ELISA reactivity, leading to the assumption that expression levels differed widely, with BVDV type 1 E2 being 100 times more reactive than the HoBi-like one. BVDV-1 antigen was diluted to 1:900, BVDV-2 to 1:150, and Hobi-like to 1:8. Any attempt to improve the signal of the latter antigen, including the use of a baculovirus expression system, has been unsuccessful so far (data not shown).

**Table 1:** Cross-neutralization titer induced in sheep by inactivated preparation of BVDV-1, BVDV-2, and HoBi-like *Pestivirus*, based on 5 independent replicates. Range of titers is reported within brackets

		SN titers	
Serum	Against BVDV-1	Against BVDV-2	Against HoBi-like
#8135 BVDV-1 immunized	**1782.89** **[1024–2048]**	48.50 [32–64]	97.01 [64–128]
#9750 BVDV-2 immunized	1.74 [0–2]	**445.72** **[256–512]**	27.86 [16–32]
#8148 HoBi-like immunized	97.01 [64–128]	776.05 [512–1024]	**2702.35** **[2048–4096]**

**Fig. 1:**
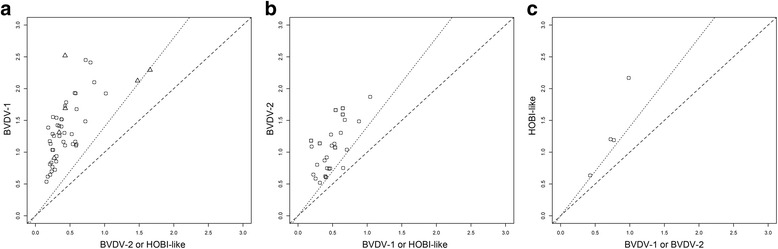
Dispersion plot of ELISA reactivity using sera from animals transiently infected or immunized with a BVDV-1 (panel **a**), BVDV-2 (panel **b**), and HOBI-like strain (panel **c**). Circles: bovine sera; triangles: rabbit sera; squares: bovine sera from experimentally infected animals. Reactivity against homologous antigen (Y axis) and the most reactive heterologous antigen (X axis) are shown. Dashed lines represent equal reactivity; dotted lines represent cut-off discrimination

In order to better evaluate the test performances, a set of 51 negative sera from 8 different farms was used to estimate the cut-off of acceptance (OD cut-off 0.5). In the other hand, sera collected from animals (bovine or rabbits) transiently infected or immunized with a defined BVDV strain were tested and found to be more reactive versus homologous BVDV antigen, although some were raised in response to different clades within the same viral species (Fig. 1). This sample set allowed us to set a discrimination cut-off at 1.42, representing the ratio between reactivity against the homologous and the heterologous antigens.

Sera from immunized sheep always reacted to a greater extent against the E2 protein corresponding to the homologous immunogen (Additional file 1: Figure S1) when used as positive controls in 17 test runs (i.e., ELISA plates). Based on the 17 ELISA runs, each control showed significantly higher reactivity against the homologous antigen as compared to the highest heterologous antigens. For each positive control serum, reactivity against the homologous antigen was compared to the highest OD obtained when the serum was tested against the heterologous antigens. The Wilcoxon signed rank test showed significant differences (*p* < 0.001 in all three cases), with the median ratio higher than 1.42 in all three (BVDV1 6.55, BVDV2 5.76, BVDV3 2.25).

**Fig. 2:**
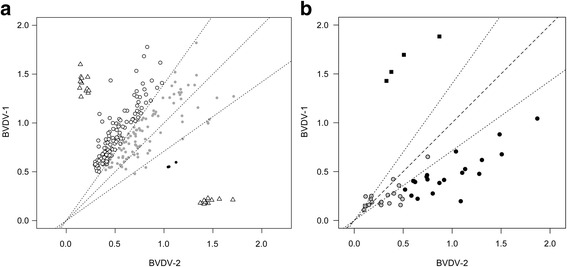
Dispersion plot of ELISA reactivity versus BVDV-1 recombinant E2 (Y axis) and BVDV-2 recombinant E2 (X axis). Diagonal and envelope lines represent equal reactivity and cut-off discrimination, respectively. Panel **a**: Reactivity of pooled milk samples. Strain specific control sera are shown as white triangles. White dots denote pool milk samples reactive against BVDV-1 antigen. Grey dots denote indeterminate samples. Black dots denote pools from a single dairy farm reactive against BVDV-2 antigen. Panel **b**: Spot test on young animals. Grey dots denote negative or indeterminate samples. Black dots denote individual serum samples reactive against BVDV-2 antigen. Black squares denote individual serum samples from a BVDV-1 positive farm

The method was then applied to a total of 436 milk pools collected from 179 dairy farms: the number of pools per farm ranged between 1 and 9. On the basis of the proposed method of classification, 33 farms showed only negative pools (*n* = 125). Further investigation revealed that no vaccination programs were in place on any of the farms during sample collection. A total of 111 pools, from 63 farms, showed the greatest reactivity against the BVDV type 1 antigen. A few milk pools from 3 farms reacted against BVDV-2 antigen. In particular, 3 pools from a single farm showed clear reactivity on E2 ELISA (Fig. 2a). Questioning farmers about their animal management practices revealed that a double-strain live vaccine, based on BVDV-1 and 2 strains, was used on the remaining 2 farms. Fourteen pools showed reactivity against HoBi-like *pestivirus* just above the level of discrimination (data not shown). The BVDV type could not be ascertained in 177 pools.

**Fig. 3:**
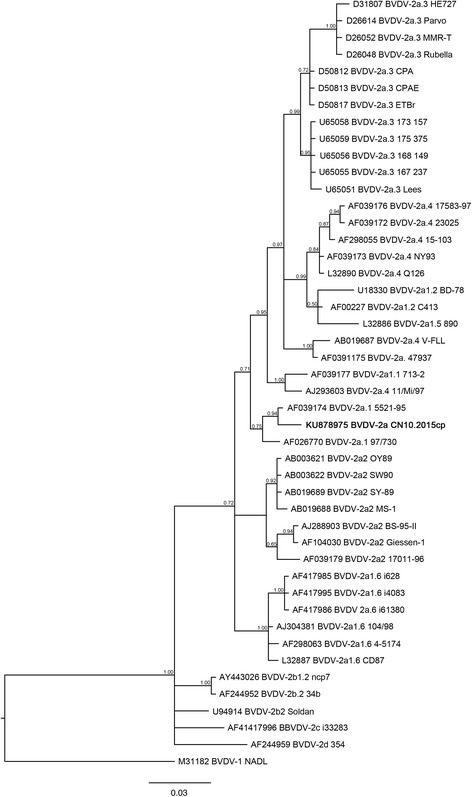
Bayesian phylogenetic tree. New taxa obtained in this study are given in bold. Posterior probability of each node is given above the branches

The farm with clear BVDV-2 reactivity was further investigated. Testing of individual young animals that had never been vaccinated confirmed that most were reactive against the type 2 antigen (Fig. 2b). Five PI animals were identified in this group. Sequences of the 5′-UTR region obtained from two of the animals were 100% similar; based on the similarity found between the newly obtained sequence and the sequences available in GenBank database and the tree topology, the analyses confirmed that the animals carried the BVDV-2a strain (GenBank accession number KU878975, Fig. 3). No PI animals were found among lactating cows.

## Discussion

Circulation of BVDV-2 in Italy was occasionally documented between 1995 and 2004 [17, 18]. Its genetic characterization accounts for less than 3% of the total pestivirus sequences retrieved from the literature. Due to its high sensitivity, RT-PCR has become a cost-effective diagnostic tool for PI identification that is applicable to pooled blood/milk samples. Sequence analyses of positive samples represent an additional cost, which is why they are rarely performed, thus limiting strain characterization at the time of diagnosis. The only genetic information available in many countries comes from periodical screening of stored PCR positive samples. In all cases, atypical or sporadic strains have been detected following PI identification. To our knowledge, this is the first immunoenzymatic assay that can differentiate BVDV species based on seroreactivity against the most variable E2 glycoprotein. The ELISA format was successfully employed on both serum samples and individual or pooled milk samples, demonstrating its use as a cost effective tool for rapid screening of dairy herds. Since pestiviruses display considerable heterogeneity within one species, we first analyzed a panel of sera raised against defined BVDV-1 and BVDV-2 strains and then evaluated the ability of each of the E2 antigens to specifically recognize homologous infection (at the species level). The results clearly suggest that discrimination is possible even for sera raised against BVDV species of different subtypes.

It should be noted that the proposed method has been roughly validated for sensitivity, specificity, and discrimination power. A recent study reported that the frequency of detection of BVDV-2 and HoBi-like viruses among 371 UTR sequences was 2.7% and 0%, respectively [19]. The identification of a single BVDV-2 positive herd in the present study is in line with a sporadic level of circulation. On the other hand, a large part of the milk pools reacted against the BVDV-1 antigen, supporting the high frequency of detection of BVDV-1. Because the E2 protein elicits neutralizing antibodies, the E2-based ELISA is unable to discriminate between vaccinated and infected animals. In this context, the greater reactivity against the type 1 antigen may also reflect the extensive use of BVDV-1 vaccination. Since a double-strain live vaccine based on BVDV-1 and 2 strains has recently been approved for the Italian market, its use may further limit the future usefulness of the proposed method for surveillance purposes. The milk pools from two farms where double-strain vaccination had been recently implemented were, in fact, slightly more reactive against the BVDV-2 antigen. Any attempt to identify circulating BVDV was unsuccessful. However, we cannot exclude that a proportion of indeterminate results (shown as grey circles in Fig. 2) may reflect the outcome of mixed seroconversion against both a BVDV-1-based vaccine and a BVDV-2 wild-type strain or against a double-strain based vaccine.

In this scenario, circulation of BVDV-1, 2, or HoBi-like *Pestivirus* may be predicted by spot tests on young, unvaccinated animals, while bulk milk ELISA may serve as a marker for herd immunity following vaccination. The serological identification of the circulation of HoBi-like *Pestivirus* is still free of confounding factors, since specific vaccines are not available, and possible seroconversion against Hobi-like antigens can be caused only by wild type viral strains. Unfortunately, the HoBi-like E2 antigen was expressed at very low levels as compared to its BVDV-1 and BVDV-2 counterparts. The lower reactivity of the strain specific HoBi-like control serum against the homologous antigen (median OD 0.75), compared to BVDV-1 and BVDV-2 control sera (median OD 1.5 against E2 BVDV-1 and E2 BVDV-2 antigens, respectively), as well as its lower discriminating power (supplementary material S1), was probably due to a lower level of expression of the transfected cells. Furthermore, we cannot exclude that the lower dilution factor (1:6) of the HoBi-like antigen during the plate coating step might also have influenced ELISA performance. For this reason, we considered as undetermined the 14 pools weakly reacting against HoBi-like antigen; this result may reflect unspecific reactivity against cellular proteins rather than against the E2 antigen (data not shown).

Sequence analysis of BVDV-2 strains isolated from PI animals belonging to the same herd indicated that the strains were all subtype 2a. This subtype was identified in northern Italy more than 20 years ago [7]. It is difficult at this stage to speculate how low levels of circulation in the absence of specific vaccination could have persisted for such a long time. The partial sequence clustered together with strains identified in the United States [20], falling into the BVDV-2a.1 clade. Unfortunately, a clear picture of the distribution and the genetic heterogeneity of BVDV-2 is not available. For this reason, we can merely hypothesize that a recent introduction through biological material and/or animal trade could be responsible for the presence of the BVDV strain identified. Extensive animal trading with European countries (mainly France and Germany) in the last 3 years was recorded for this herd, further supporting the role of this risk factor in the introduction of unusual *Pestivirus*es. It is noteworthy that the herd has been repeatedly vaccinated with the BVDV-1-based inactivated vaccine, so that antibody reactivity of lactating cows against the homologous E2 glycoprotein could be expected. Though vaccination may represent a bias when interpreting the data, outbreaks of infection with different BVDV species have occurred, given the strong immune response the infected animals developed, especially under the infective pressure of the PI animals.

## Conclusions

This study provides evidence for how serological surveillance of BVDV can provide a valuable tool for the prompt identification of atypical *Pestivirus*. Serological screening to differentiate BVDV infections can be used for monitoring the introduction and circulation of different *Pestivirus* species. Moreover, serological investigation is a very easy way to follow and control vaccination protocols, especially if different BVDV species are used as the vaccine strain. This approach can improve the surveillance system and help identify possible risk factors associated with atypical BVDV infections.

## Methods

### Viruses and cells

The BVDV-1 NADL (courtesy of Dr. Ferrari, Istituto Zooprofilattico Sperimentale di Lombardia ed. Emilia Romagna, Brescia, Italy), BVDV-2232/02 [17], and HoBi-like *Pestivirus* Italy-83/10cp [21] cytopathogenic strains were used for immunization procedures (as inactivated preparations) and for the virus neutralization (VN) assays to assess the VN titer in immunized animals. All viruses were propagated in pestivirus-free Madin-Darby bovine kidney cell lines (MDBK; ATCC CCL-22), cultured in Dulbecco’s modified essential medium (DMEM) (Sigma-Aldrich, Schnelldorf, Germany) containing 10% fetal bovine serum (FBS, Gibco, Thermo Scientific, Rodano, Italy, tested negative for pestivirus or anti-BVDV antibody), 2 mM of L-glutamine, 100 IU/ml of penicillin (Sigma-Aldrich), 100 mg/ml of streptomycin (Sigma-Aldrich), and 2.5 mg/ml of amphotericin B. Cells were incubated at 37 °C with 5% CO_2_. Human embryo kidney cell lines (HEK293T; ATCC, CRL-1573) were cultured following the same procedure and used for transfection experiments.

For immunization, viral suspensions were inactivated with 0.05% β-propiolactone and emulsified initially with Tween-80 (4.1% *v*/v) and then with a mixture (50% v/v) of Montanide ISA 563 (Seppic Inc., Paris, France), Marcol 52 (Esso Italiana S.r.l., Rome, Italy), and Montane 80 (Seppic Inc.) in a 30:63:7 ratio, as previously described [22].

### Strain specific sera

To obtain strain-specific control sera in the ELISA procedure, three female, *Biellese* breed sheep were selected from the *Pestivirus*-free flock belonging to the Veterinary Teaching Hospital at the Department of Veterinary Science, University of Torino. The animals were immunized with the inactivated preparations described above. Each sheep received two inoculations of 2 ml administered 28 days apart. All the animals were housed at the Veterinary Teaching Hospital under standard husbandry conditions. The blood was drawn on day 42 after anesthesia followed by euthanasia. Experiments were carried out at the Veterinary Teaching Hospital laboratories and in compliance with the relevant national legislation on experimental animals and animal welfare (Italian Ministry of Health DL 116/92), upon authorization by the competent authority (Italian Ministry of Health Aut Req 26,062,013). Virus neutralization (VN) titer against homologous and heterologous immunogens was determined for each serum by cross-seroneutralization (5 technical replicates for each serum). Sera were collected and freeze-dried in 5-ml aliquots for long-term storage.

BVDV negative samples were collected from 8 BVDV-free farms for setting the acceptance cut-off. All the samples showed negative results when tested with a commercial IDEXX Ab ELISA test. In addition, sera collected from animals transiently infected with a defined BVDV strain were retrieved from previous studies. The sample set included 44 sera from 3 BVDV-1b positive farms, 30 sera from a BVDV-2a positive farm, 6 sera from a BVDV2c experimental infection (CODA-CERVA, Belgium), and 4 sera from a BVDV-3 positive farm [2].

To take into account the considerable heterogeneity even within one species, such as BVDV 1, sera from rabbits immunized with different members of the clade (BVDV1b, d, g, h, and e) were kindly provided by Gian Mario Demia (Istituto Zooprofilattico Sperimentale di Umbria e Marche, Perugia, Italy). Strain-specific sera were used to identify the best parameters for interpretation of the results.

### Gene fragment, cloning and eukaryotic expression

**Fig. 4:**
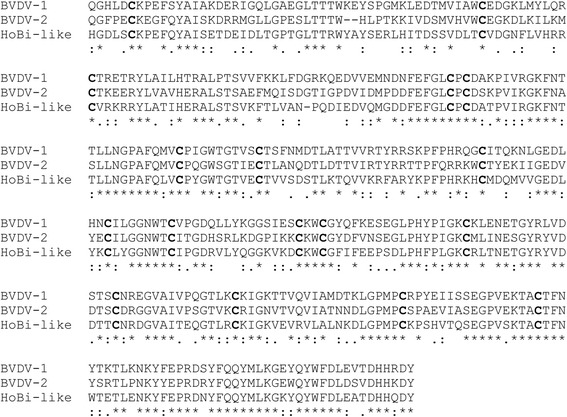
Amino acid alignment of BVDV-1, BVDV-2 and HoBi-like E2 glycoprotein ectodomains expressed in recombinant form in the present study. Conserved cysteine residues are given in bold. Asterisks denote identical amino acids, colons (:)conserved substitutions, and dots (.)semi-conserved substitutions

Amino acid sequences, corresponding to the ectodomain of the E2 protein of BVDV-1 (strain NADL), BVDV-2 (strain 1373), and HoBi-like *Pestivirus* (Italy-83/10cp) were retrieved from the GenBank database (accession number CAB91847, AAD38683 and AFL65619.1). Synthetic genes (Eurofins MWG, Ebersberg Germany) were constructed carrying HindIII and XhoI restriction sites to facilitate unidirectional cloning. Genes were subcloned into pSecTag2/Hygro plasmids (Invitrogen, Waltham, MA, USA), allowing the extracellular sorting and secretion in the medium of transiently transfected mammalian cells. These plasmids were used to transform chemically competent *Escherichia coli* (strain BL21) by heat shock. Ampicillin-resistant colonies were subjected to PCR for rapid screening and sequencing to confirm the authenticity and in-frame insertion of each fragment. Endotoxin-free plasmid purification from 500 ml of LB culture was carried out using NucleoBond® Xtra Midi/Midi Plus (Macherey-Nagel, Düren, Germany). Sub-confluent human embryonic kidney (HEK293T) cells, cultured in 75-cm^2^ flasks, were transfected with 9 μg of plasmid and 21 μl of LTX transfection reagent (Thermo Fisher Scientific, Waltham, MA, USA) in 6 ml of DMEM [23]. After 6 h at 37 °C with 5% CO_2_, the transfection medium was replaced with 6 ml of protein-free medium (ExCell293, Sigma-Aldrich) and the flasks were incubated as described above for an additional 42 h. The medium was then collected, centrifuged at 5000 g for 10 min to remove cell debris, and stored at −80 °C until use. The amino acid sequences of the three E2 proteins used in the present study are shown in Fig. 4.

### Farms, milk and serum samples

During a previous study on infectious bovine rhinotracheitis, milk samples were collected from 179 dairy farms located in the Province of Cuneo, Piedmont, in collaboration with the Regional Farmers’ Association (Associazione Regionale Allevatori, ARA). Written consent of the milk collection for research purposes for was obtained from all farmers. During that study, individual milk samples were collected from each farm, forming a total of 436 milk pool samples from about 35 lactating animals. The same samples were used for the present work. At the time of sample collection, the BVDV status and vaccination programs for the dairy farms were unknown.

Following milk testing, we performed additional testing on one of the farms that had displayed greater reactivity against the E2 antigen of BVDV-2. Individual blood and milk samples were collected from young unvaccinated animals and lactating cows, respectively, and further analyzed by spot test (5–10 individual tests per group) and for PI identification [16].

### ELISA procedure

All control sera were normalized to a dilution corresponding to a VN titer of 430. Due to the different levels of expression of the three E2 proteins, the optimal dilution of each antigen was carried out using control sera by checkerboard titration. Antigens were diluted in 0.1 M carbonate/bicarbonate buffer, pH 9.6, and used to coat Nunc Maxisorp plate wells overnight at 4 °C.

After blocking non-specific sites, the milk or serum samples were assayed undiluted or diluted 1/20 in PBS 1.25% casein, respectively. Samples were incubated for 1 h (blood serum and controls) or 2 h (milk) at room temperature. After washing, peroxidase labeled protein G, diluted to 10 ng/ml in PBS 1.25% casein, was added to the wells and the plates were incubated for 45 min at room temperature. After the final wash, the reaction was developed with 3,3′,5,5′-tetramethylbenzidine (TMB) and stopped with 0.2 M H_2_SO_4_. Reactivity of the monospecific control sera against the three antigens was evaluated in 10 independent tests and classified depending on the immunogen (supplementary material S1).

Negative sera (*n* = 28) were from animals coming from a not vaccinated farm where all the animals resulted negative to official diagnostic tests. All the samples were tested against the three antigens, and the cut-off for considering unknown sera as positive (i.e., valuable for viral typing) was identified as the mean reactivity plus five standard deviations (mean reactivity 0.145, standard deviation 0.058). Samples showing reactivity ≥ 0.5 (corresponding to a VN titer between 8 and 16) were investigated for further typing.

Sera collected from animals transiently infected or immunized with a defined BVDV strain were then tested against the three antigens. The sample set included 44 sera from 3 BVDV-1b positive farms, 30 sera from a BVDV-2a positive farm, 6 sera from animals experimentally infected with BVDV2c strain, and 4 sera from a BVDV-3 positive farm. We applied a BVDV typing method to analyze the data. The ratio between the homologous antigen and the most reactive heterologous antigen was calculated for each sample. The distribution of ratios was evaluated (Shapiro-Wilk test for normality assumption) and the mean minus 1 standard deviation was considered as the discrimination cut-off for antigen reactivity. This approach indicated that 1.42 is the most restrictive ratio for seropositivity discrimination among antigens. In other words, a sample is considered positive for a given antigen if the optical density (OD) against that antigen is greater than 142% as compared to the ODs obtained against the other antigen with the highest reactivity.

### PI identification

PI animals were identified using an antigen ELISA (IDEXX BVDV Ag/Serum Plus, IDEXX, Westbrook, ME, USA). Two of the 5 PI animals (CN10.2015.813 and CN10.2015.821) were selected for viral strain characterization and blood serum was collected from both. The first animal died after developing MD during the study, and samples from the spleen and intestinal epithelium were collected. Tissue homogenates and blood serum from the animal were used to isolate the viral strain on MDBK culture. Viral RNA was extracted from homogenized tissues and infected cell culture supernatant using an EZ1 Virus kit v2.0 (Qiagen, Hilden, Germany). For each sample, the 5’UTR region was amplified as previously described [5]. Amplicons were purified using Nucleospin Gel and PCR Clean-up kit (Machery Nagel) and directly sequenced in both directions (BMR Genomics, Padua, Italy). Consensus sequences were aligned to homologous reference sequences available in the online databases (Clustal W algorithm embedded in Geneious software ver. 9.0.2), referring to the BVDV-2 classification proposed previously [24]. Phylogenetic analysis was conducted by selecting the best evolutionary model with jModelTest version 2.1.7 [25] and applying the Bayesian heuristic search approach included in MrBayes software [26].

## Additional file

Additional file 1: Figure S1. Reactivity of monospecific sera against the three different recombinant antigens. Optical density values are reported on the y axis. (TIFF 81 kb)

## Abbreviations

BDV: Border disease virus
BVDV: Bovine viral diarrhea virus
CP: Cytopathic (strain)
DMEM: Dulbecco’s modified essential medium
ELISA: Enzyme-linked immunosorbent assay
FBS: Fetal bovine serum
MD: Mucosal disease
NCP: Non cytopathic (strain)
OD: Optical density
PBS: Phosphate-buffered saline
PI: Persistently infected (animal)
TMB: 3,3′,5,5′-tetramethylbenzidine
VN: Virus neutralization (titer)
