# Supplementary figures and images for: Pestivirus infection in cattle dairy farms: E2 glycoprotein ELISA reveals the presence of bovine viral diarrhea virus type 2 in northwestern Italy
Source: BMC Vet Res. 2017 Dec 4;13:377. doi: 10.1186/s12917-017-1305-z (PMC5715619; doi:10.1186/s12917-017-1305-z)

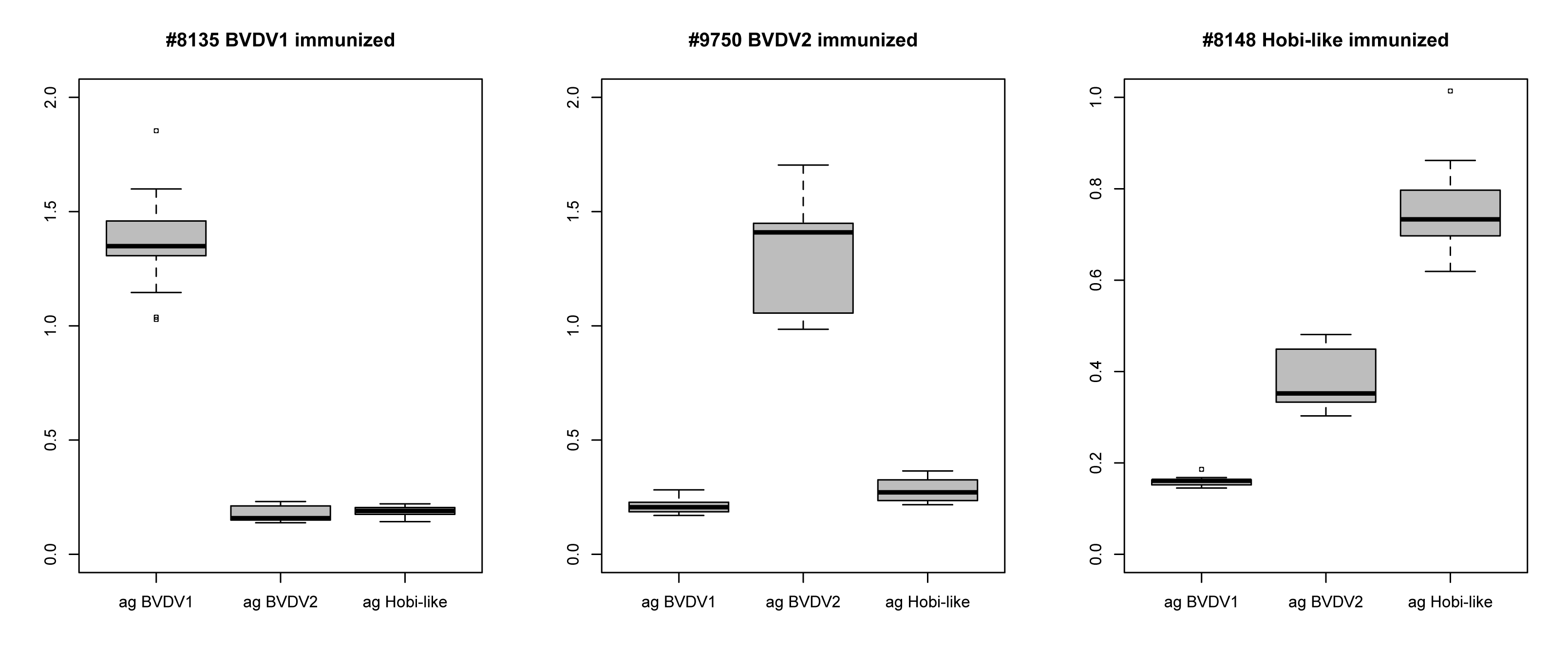

Supplement: Additional file 1: Figure S1. — Reactivity of monospecific sera against the three different recombinant antigens. Optical density values are reported on the y axis. (TIFF 81 kb) [file 12917_2017_1305_MOESM1_ESM.tif]
